# Anderson-Fabry disease cardiomyopathy: an update on epidemiology, diagnostic approach, management and monitoring strategies

**DOI:** 10.3389/fcvm.2023.1152568

**Published:** 2023-06-02

**Authors:** Tauben Averbuch, James A. White, Nowell M. Fine

**Affiliations:** ^1^ Division of Cardiology, Department of Cardiac Sciences, University of Calgary, Calgary, AB, Canada; ^2^ Stephenson Cardiac Imaging Center, Alberta Health Services, Libin Cardiovascular Institute, Cumming School of Medicine, University of Calgary, Calgary, AB, Canada

**Keywords:** anderson-Fabry disease, heart failure, cardiac imaging, management, enzyme replacement therapy

## Abstract

Anderson-Fabry disease (AFD) is an X-linked lysosomal storage disorder caused by deficient activity of the enzyme alpha-galactosidase. While AFD is recognized as a progressive multi-system disorder, infiltrative cardiomyopathy causing a number of cardiovascular manifestations is recognized as an important complication of this disease. AFD affects both men and women, although the clinical presentation typically varies by sex, with men presenting at a younger age with more neurologic and renal phenotype and women developing a later onset variant with more cardiovascular manifestations. AFD is an important cause of increased myocardial wall thickness, and advances in imaging, in particular cardiac magnetic resonance imaging and T1 mapping techniques, have improved the ability to identify this disease non-invasively. Diagnosis is confirmed by the presence of low alpha-galactosidase activity and identification of a mutation in the GLA gene. Enzyme replacement therapy remains the mainstay of disease modifying therapy, with two formulations currently approved. In addition, newer treatments such as oral chaperone therapy are now available for select patients, with a number of other investigational therapies in development. The availability of these therapies has significantly improved outcomes for AFD patients. Improved survival and the availability of multiple agents has presented new clinical dilemmas regarding disease monitoring and surveillance using clinical, imaging and laboratory biomarkers, in addition to improved approaches to managing cardiovascular risk factors and AFD complications. This review will provide an update on clinical recognition and diagnostic approaches including differentiation from other causes of increased ventricular wall thickness, in addition to modern strategies for management and follow-up.

## Introduction

Anderson-Fabry disease (AFD) is an X-linked glycogen storage disorder, characterized by the accumulation of globotriaosylceramide (Gb3) due to a deficiency of α-galactosidase. First described independently 1898 by Johannes Fabry and William Anderson in two patients with angiokeratoma (1), the GLA gene responsible for the production of α-galactosidase was identified in 1974 (1). Over time, AFD has been identified as a multi-system disease, with characteristic cardiac, renal, neurologic, dermatologic, and gastrointestinal manifestations. Additionally, the variable presentation of AFD has increasingly been recognized, with both classical and non-classical (or late onset) phenotypes (2). Patients with classical AFD are more commonly males in early adulthood who present with painful peripheral neuropathy, cutaneous lesions, and gastrointestinal upset in childhood or the early teenage years, with gradual development of heart failure (HF) and chronic kidney disease (3). Late-onset AFD is considerably more variable, presents in middle-aged or older patients, may be limited to cardiac or renal involvement (4) and has been described as a more common presentation for affected female patients. In contrast to classical AFD, α-galactosidase levels are higher in late-onset AFD, accounting for the delayed presentation (5).

Patients with AFD face significant morbidity and mortality, with reduced life expectancy. Early diagnosis of AFD is essential in order to start therapy, however, the diagnosis of AFD is frequently delayed by several years after the development of symptoms (6). Treatment has historically been limited to enzyme replacement therapy with recombinant α-galactosidase, however, novel therapies are also now available with new agents in development (7). In this comprehensive review, we describe the genetic basis of AFD and its pathophysiology, as well as the presentation and natural history of AFD with a focus on sex-based on phenotypic differences and a particular emphasis on the natural history of cardiac involvement. We review the diagnostic process in AFD, as well as non-invasive techniques to differentiate AFD from mimics, such as hypertrophic cardiomyopathy. Lastly, we review the evidence for enzyme replacement therapy in AFD as well as novel therapeutic agents.

## Pathophysiology and epidemiology

### Pathophysiology

**Figure 1:**
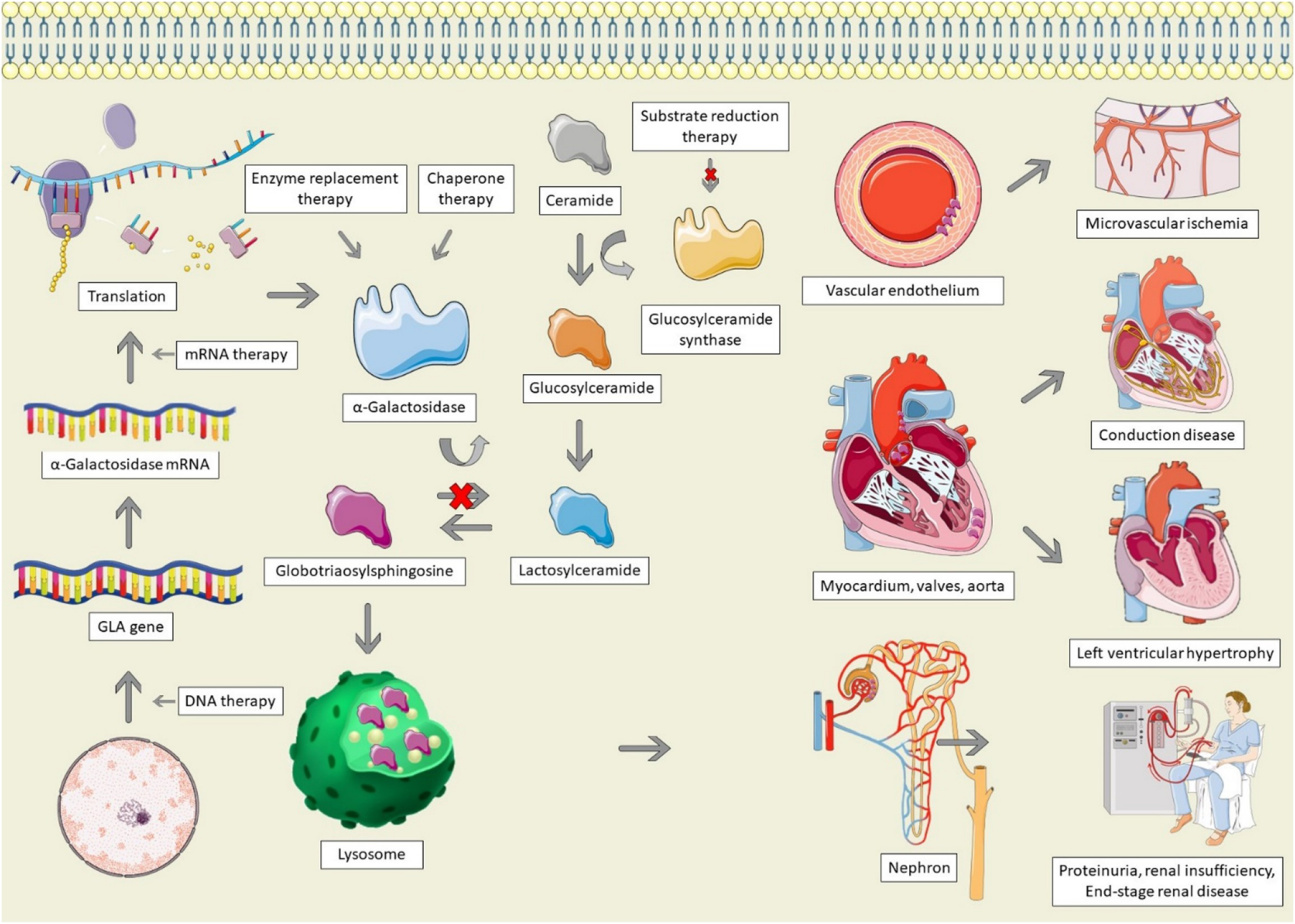
Pathophysiology and therapeutic options for Anderson-Fabry disease. AFD is characterized by GLA mutations, resulting in absent, insufficient, or dysfunctional α-galactosidase. Alpha-galactosidase catalyzes the conversion of globotriaosylsphingosine (Gb3) to Lactosylceramide; in its absence, Gb3 accumulates in lysosomes, particularly within the vascular endothelium, cardiac structures, and the nephron, leading to end-organ dysfunction. Therapeutic options for AFD involve increasing the production of α-galactosidase, replacing it, stabilizing misfolded galactosidase, or decreasing the production of glucosylceramide. The Figure was partly generated using Servier Medical Art, provided by Servier, licensed under a Creative Commons Attribution 3.0 unported license, as well as from freepik.com (https://www.freepik.com/free-vector/animal-cell-anatomy_26763764.htm).

Anderson-Fabry disease (AFD) is caused by mutations of the GLA gene, located at Xq22 on the X chromosome (8). Mutations result in a deficiency or absence of the enzyme α-galactosidase, resulting in the accumulation of globotriaosylceramide (Gb3), which is trafficked to the lysosomes in numerous tissues, including the heart, kidneys, skin, and vascular endothelium (Figure 1) (8). In female patients, random inactivation of the X-chromosome can lead to residual α-galactosidase activity, with variable phenotypic presentation. Deposition of Gb3 into the vascular endothelium is a significant contributor to the end-organ manifestations of AFD (9). Gb3 binds to lipoprotein receptors on the vascular endothelium, where it is then deposited within the vascular intima and media. Binding of Gb3 leads to increased leukocyte migration and adhesion, leading to the production of pro-inflammatory as well as vascular smooth muscle hypertrophy (10). This process ultimately leads to reactive oxygen species production and a decrease in nitric oxide, resulting in endothelial injury and dysfunction (11). The inflammatory cytokine production also contributes to a pro-thrombotic state (12). Vascular smooth muscle proliferation and microthrombi of the intramural coronary arterioles leads to reduced flow reserve and can ultimately cause infarction and fibrosis, whereas involvement of the cerebral vasculature contributes to the high risk of stroke in AFD (13, 14).

Infiltration of Gb3 into the heart can lead to left ventricular hypertrophy (LVH), valvular disease, systolic and diastolic dysfunction, and conduction system disease (15). LVH is not solely caused by deposition of Gb3 (16). The deposition of Gb3 leads to a cascade of inflammation and oxidative stress, leading to extracellular matrix remodelling and hypertrophy, culminating in myocyte necrosis and fibrosis (17). In a study in which plasma from AFD patients was administered to rat vascular smooth muscle cells and neonatal mouse cardiomyocytes, there was a significant increase in cellular proliferation, which was correlated with the left ventricular mass index of the Fabry patient, suggesting that a circulating factor drives LVH in AFD (18). Similarly, another study found that patients with AFD have higher levels of sphingosine-1-phosphate (S1P) (19). Administration of S1P to mice led to cardiac remodelling resembling AFD, as well as increased vascular smooth muscle proliferation in a dose-dependent manner, suggestive that S1P may be one of the circulating factors responsible for LVH in AFD (19). Additionally, small vessel hypertrophy and Gb3 deposition can lead to microvascular ischemia, causing severe diastolic dysfunction, contributing to LVH (20). Lastly, hypertension is common among AFD patients, which may result from activation of the renin-angiotensin-aldosterone system due to direct renal injury, further contributing to LVH (21).

To date, more than 1,100 mutations of the GLA gene associated with development of AFD have been identified (22). Mutations can be classified as pathological, likely pathological, or variants of unknown significance (22). The type of mutation can be missense (23), nonsense (24), or frame shift (25); although the majority of documented mutations are missense (23). While several mutations have been identified, there does not appear to be one dominant mutation; the mutations observed appear to be limited to individuals and families (26). Mutations typically affect active binding sites of the GLA protein, or impaired trafficking to the lysosome, leading to early degradation within the endoplasmic reticulum (26). In addition to causing loss of function of the GLA protein, missense variants may also interfere with the wild-type product through dominant negative effects (24). The specific mutation involved has implications for the natural history of AFD in each patient, as well as the relevant organ systems involved.

Classical AFD is early-onset, with characteristic multi-system involvement, including cardiac, renal, and dermatologic manifestations. However, later onset AFD may predominantly affect a single system, as in the case of cardiac variants. It can be challenging to predict the clinical manifestations of a given genotype. However, in a population of Italian AFD patients, a decision tree model was derived to predict classical or variant AFD, based on the sequential and structural properties of the mutation (27). In a separate study of Chinese AFD patients, among males, patients with frameshift and nonsense mutations had a classical phenotype, while missense mutations could lead to classical or variant phenotypes (28). Among female patients, the majority of patients with frameshift mutations had a classical phenotype (28). Prediction tools for specific organ involvement for a given mutation have yet to be developed. Cardiac specific variants of AFD are being increasingly recognized (29, 30). In order to address the challenge for correlating genotype with phenotype, the AFD Genotype-Phenotype Working Group developed a 5-stage iterative system for previously unassigned GLA variants, which involved clinical assessment of affected patients, published literature, expert consensus, and a 2-point scoring system, with final validation by Kaplan-Maier curve analysis for severe event-free survival (31). The 2-point GLA scoring system is based on the proportion of males with the GLA variant who have either angiokeratomas or cornea verticillata, and has been shown to discriminate between classic and late-onset AFD (31). Using this iterative process, 32 of 33 previously unclassified GLA variants were determined to be pathogenic, which led to classification of previously unclassified GLA variants as pathogenic in 17.5% and 16.6% of male and female patients, respectively (31).

### Epidemiology

The overall prevalence of AFD causing clinical disease is estimated as 1 in 40,000 to 1 in 170,000 births, affecting all ethnic groups, with specific ethnic predispositions (32). The prevalence of mutations known or thought to be related to the development of AFD is considerable higher, although the proportion of individuals with GLA variants who develop clinical disease is unknown. In a study of the 200,643 individuals in the UK Biobank, the prevalence of likely pathogenic late-onset variant mutations was 1 in 5,732, and 1 in 200,643 for mutations causing classic AFD (33). Similarly, studies of the prevalence of GLA variants in newborn screening programs have found a high prevalence of disease-causing mutations, ranging from 1 in 1,250 male newborns in Taiwan to 1 in 21,973 in the USA (34, 35). Overall, later-onset variants were more common in studies involving newborn screening; the ratio of late-onset to classic variants ranged from 7:1 in Italy to 16:1 in Taiwan (2). While mutations associated with late-onset variant AFD are more common than mutations causing classical AFD; classical AFD is more common in cohorts of patients with established disease, suggesting that late-onset variant AFD is significantly underrecognized (36). The prevalence of AFD within specifically selected clinical populations with manifestations of AFD is considerably higher. Among patients with LVH, two recent cohort studies found a prevalence of AFD of 0.9% and 0.3% (37, 38). The prevalence of AFD in otherwise unexplained LVH is higher, ranging from 2% in Canada and Hong Kong (39) to 4% in the Czech Republic (40).

## Clinical presentation and natural history

AFD is classically a multisystem disease with prominent cardiac involvement, including LVH, systolic and diastolic dysfunction, valvular disease, and small vessel ischemia. Although male and female patients may present with similar symptoms and systems involved, the proportion affected and age at development of each manifestation differs (Table 1). In the largest cohort study of AFD, the mean (SD) age of symptom onset was 9 (13.2) for male and 13 (19.1) for female patients, while the median age at diagnosis was 23 and 32, respectively (41). Cardiac symptoms at presentation were similar between male and female patients (13% vs. 10%), but the median age of onset was far younger for male than female patients (12 vs. 32 years) (41). Cardiovascular events, defined as arrhythmia, myocardial infarction, angina, HF, or requirement for a cardiac procedure were more common in male than female patients (19% vs. 14%) and occurred at a lower median age (41 vs. 47) (41). While cardiac symptoms were not as common at diagnosis, the prevalence of cardiac involvement overall is considerably higher. In the Fabry Outcome Survey, 61.4% of male and 46.3% of female patients with AFD had cardiac symptoms (42), including dyspnea (23.1%), angina (22.4%), palpitations (26.5%), and syncope (3.2%) (42).

**Table 1:** Natural history of symptoms, signs, and complications of Anderson-Fabry disease.

Parameter	Prevalence		Age of onset	
Symptoms	Male	Female	Male	Female
**Cardiac**				
Angina	16.0 (173)–27.8 (174)	14.9 (175)–56.5 (174)	15.4 (176)–48.6 (174)	17.3 (176)–41.5 (175)
Palpitations	20.3 (177)–28.0 (173)	20.2 (175)–47.5 (178)	13.7 (176)–35.7 (177)	41.6 (175)–44.6 (42)
Shortness of breath	23.2 (42)–36.0 (173)	20.0 (173)–22.8 (42)	36.9 (42)–39.9 (42)	42.4 (42)–45.8 (42)
**Neurologic**				
Neuropathic pain	66.7 (176)–81.4 (179)	41.0 (41)–83.1 (180)	7.6 (176)–9.0 (41)	10.0 (41)–29.1 (180)
**Gastrointestinal**				
Abdominal pain	23.2 (181)–44.4 (176)	11.8 (181)–52.6 (182)	5.0 (181)–10.0 (183)	9.5 (181)–14.0 (41)
Diarrhea	26.8 (179)–33.3 (176)	18.5 (175)–46.0 (182)	5.0 (181)–9.9 (183)	9.5 (181)–19.0 (175)
**Signs and complications**				
Left ventricular hypertrophy	46.0 (184)–73.1 (45)	19.0 (45)–38.0 (48)	38.0 (184)–57 (45)	50.4 (175)–73 (45)
Any AV block	20.3 (45)	10.3–11.3 (45)	n.d.	n.d.
Complete AV block	12.7 (45)	1.6 (45)	60.0 (45)	79.0 (45)
Ventricular tachycardia or fibrillation	11.6 (173)–14.1 (45)	0.0 (173)–20.0 (185)	38.0 (173)–57.0 (45)	n.d.
Atrial fibrillation	7.6 (45)–18.8 (173)	2.4 (45)–18.8 (173)	38.0 (173)–67.0 (45)	50.0 (173)–77.0 (45)
Heart Failure	3.5 (186)–55.8 (187)	2.3 (186)–28.1 (187)	36.9 (42)–64 (45)	42.4 (42)–76 (45) v
Aortic root dilatation	32.7 (69)	5.6 (69)	46.8 (69)	69.1 (69)
Ascending aortic dilatation	29.6 (69)	21.1 (69)	48.8 (69)	>50 (69)
Valvular disease (any)	48.6 (64)	38.1 (64)	32.3 (42)–54 (64)	31.7 (42)–54 (64)
Moderate to severe regurgitant disease	5 (64)	5 (64)	n.d.	n.d.
Severe stenotic disease	0 (64)	0 (64)	n.d.	n.d.
Proteinuria	44.0 (184)–52.6 (45)	21.0 (45)–45.2 (166)	13.8 (176)–55.0 (45)	14.1 (176)–51.0 (45)
End-stage renal disease	2.4 (188)–13.7 (189)	1.2 (175)–2.0 (189)	38.0 (189)–55.0 (190)	38.0 (189)–40.0 (175)
Cerebrovascular disease (stroke or TIA)	6.9 (191)–48.0 (192)	4.3 (191)–31.7 (192)	28.8 (184)–46.0 (192)	38.8 (175)–52.0 (192)

AV, Atrioventricular; TIA, Transient Ischemic Attack.

The life expectancy of patients with AFD is significantly reduced. Within registry studies of AFD, the mean age at death ranged from 51.8 to 54.3 years for male patients and 62.0–64.4 years for female patients, with 36%–53% of deaths as a result of cardiac disease (43). In the Fabry Outcome Survey, LVH was present in 69% of deceased patients; conduction disease in 45%, and valvular disease in 38% (45). The life expectancy for male patients in the Fabry Registry was estimated as 58.2 years, while for female patients was 75.4 years (44). In the Fabry Registry, deceased patients were diagnosed later in life, at a mean age of 39.8 vs. 24.4 years for male and 55.4 vs. 32.7 years for female patients, and had only received enzyme replacement therapy for a median of 12 vs. 4 months for male and female patients, respectively (44). The short life-expectancy in this registry likely reflects delays in diagnosis and therapy; the life-expectancy of contemporary patients receiving early administration of enzyme replacement therapy has yet to be determined but is likely much higher.

Characterization of outcomes in late-onset AFD is less well-established. In a study of 203 patients with late-onset AFD due to the p.F113l GLA mutation, the mean (SD) age at diagnosis was 49 (15) for male patients and 44 (19) for female patients (45). LVH was found in 73.1% of male vs. 19.0% of female patients, and increased with age; all male patients older than 60 and all female patients older than 80 had LVH (45). HF was more common in male than female patients (32.9% vs. 14.8%), while mean survival free from HF was higher for female than male patients (64 vs. 76 years) (45). The late-onset phenotype of AFD appears to present with a greater burden of cardiovascular manifestations and longer life expectancy.

## Cardiac involvement in AFD

LVH is the most common cardiac manifestation of AFD and is an important determinant of clinical outcomes. In a contemporary study of 560 patients treated with enzyme replacement therapy, 55% of patients had LVH at baseline (46). LVH has been found to correlate with α-galactosidase activity levels; consequently, male patients more frequently have LVH than female patients (47). LVH develops early in the course of classic AFD; in a longitudinal study of 76 patients, LVH was present in 11.1% of patients between the age of 20–29, 40.6% of patients between 30 and 39, and 76.5% of patients between 50 and 59 (48). Male patients experienced a greater increase in left ventricular mass index over time compared to female patients (4.07 ([1.03] vs. 2.13 [0.81]g/m^2.7^ per year) (48). While LVH is common in both classical and non-classical AFD, left ventricular mass index is higher in classic AFD (36).

While concentric LVH is the most common pattern of hypertrophy, a variety of patterns have been described, including concentric, apical, septal, and eccentric (49). In a study of 166 patients with AFD the pattern was concentric remodeling in 21.6%, concentric LVH is 30.9%, eccentric LVH in 5.2%, and asymmetrical septal hypertrophy in 6.2% (48). Left ventricular outflow tract obstruction is infrequently described in AFD due to asymmetric septal hypertrophy or systolic anterior motion of the mitral valve; AFD may be identified in the context of evaluation of hypertrophic cardiomyopathy (50). In studies of patients initially diagnosed with hypertrophic cardiomyopathy, the prevalence of AFD ranged from 0.5 to 1.0% (29, 51). A recent case series has also identified left ventricular mid-cavitary obstruction due to papillary muscle hypertrophy as an infrequent but significant manifestation of advanced AFD (52).

### Conduction system disease, dysrhythmias, and sudden cardiac death

Deposition of glycosphingolipids into the conducting fibers of the heart can lead to conduction system disease and dysrhythmias. In a human stem-cell model of AFD, AFD cardiomyocytes had enhanced sodium and calcium channel function, with higher spontaneous action potential frequency and shorter action potential duration (53). Similarly, electrophysiologic studies of conduction tissue from AFD patients found shorter conduction and a prolonged refractory period; additionally, the burden of conduction tissue infiltration correlated with the burden of conduction disease and arrhythmia (54). Patients with AFD are at particular risk of atrial fibrillation due to both left atrial dilatation and dysfunction (55). While left atrial dilatation in AFD can develop as a consequence of left ventricular diastolic dysfunction and LVH, the development of left atrial dilatation is independent of LVH or left ventricular diastolic dysfunction, and often predates LVH (56).

In a cohort study of patients with late-onset AFD due to the p.F113l GLA mutation, atrial fibrillation occurred in 4.4% of patients who underwent Holter monitoring, while non-sustained ventricular tachycardia (VT) occurred in 14.1% of male and 5.6% of female patients which increased in frequency with age, such that 33.3% of both male and female patients older than 70 years had non-sustained VT (45). The mean (SD) age of onset for atrial fibrillation was 67 (11) for male patients and 77 (3) years for female patients (45). Any degree of atrioventricular (AV) block was present in 20.3% and 10.3% of male and female patients (45). Male patients more commonly required a permanent pacemaker (12.7% vs. 2.4%) or implantable cardioverter-defibrillator (1.3% vs. 0.8%) than female patients (45). Complete AV block developed at a mean (SD) age of 60 (7) for male patients and 79 (6) years for female patients. Patients with AFD also face an increased risk of sudden cardiac death (SCD): in a systematic review and meta-analysis, SCD was the cause of cardiac death in 62% of cases (57). Risk factors for SCD included male sex, increasing age, LVH, non-sustained VT, and late-gadolinium enhancement on cardiac magnetic resonance imaging (CMR) (57).

### Diastolic and systolic dysfunction

AFD is associated with abnormalities in both diastolic and systolic function, due to a combination of direct infiltration of the myocardium by glycosphingolipids, fibrosis, and microvascular ischemia, and may result in overt, symptomatic heart failure. The prevalence of systolic dysfunction in AFD varies depending on the method of assessment; overt systolic dysfunction with reduced left ventricular ejection fraction (LVEF) is uncommon; in a cohort study from 2010, the prevalence of LVEF < 55% was 6.8% (47); the prevalence has not been reassessed in more recent cohort studies. Global longitudinal systolic strain (GLS), a more sensitive marker of systolic function, is frequently abnormal in AFD. In a recent study of 160 patients with AFD, patients with AFD had worse mean [SD] GLS than controls, particularly among those with LVH (−16.5%[4.2] vs. −21.2% [2.7]), with no differences between sexes (58). Patients with AFD without LVH had worse longitudinal strain at the mid to apical, anterior, and inferolateral left ventricular walls relative to controls (58). GLS may allow for the identification of early disease progression in AFD prior to the onset of overt LVH. Similarly, strain imaging can identify early right ventricular involvement; in one study right ventricular systolic function by standard echocardiographic assessment was reduced in 8%, while 3- or 6-segment right ventricular strain was reduced in 40%, with worse right ventricular strain in patients with LVH than those without (59). Comparatively, diastolic dysfunction is significantly more common than systolic dysfunction in AFD. In one study, diastolic dysfunction was found in 43.8% overall, and higher in patients with late gadolinium enhancement on CMR than those without (moderate: 28.1% vs. 2.1%, severe: 14.0% vs. 0%) (60). Diastolic dysfunction may develop with or without LVH, and is an important contributor to the development of left atrial enlargement and atrial fibrillation (15). While overt, symptomatic HF is less common than abnormal strain, an elevation in amino-terminal pro-B-type natriuretic peptide (NT-proBNP) has been found in 57% of patients with AFD, and correlates with diastolic dysfunction (61, 62).

### Ischemic heart disease

AFD is associated with small rather than medium or large-vessel ischemia (20). Small vessel ischemia is common in AFD. In one cohort study of patients with HCM and AFD, myocardial infarction with non-obstructive coronary arteries (MINOCA) occurred in 7.5% of AFD patients compared to 0.5% of HCM patients over a mean follow-up of 4.5 years (63). Furthermore, MINOCA was more common among patients with LVH, with MINOCA occurring in 17.7% of patients with LVH compared with 1.7% of patients without LVH (63). AFD was independently associated with MINOCA (OR 6.12) suggesting that MINOCA may represent a red flag for AFD (63).

### Valvular and aortic disease

AFD is associated with the development of valvular stenosis and regurgitation, although valvular heart disease is generally uncommon. Regurgitant lesions are thought to develop due to glycosphingolipid deposition within the valve leaflets and sub-valvular apparatus, as well as geometric distortion of the atria, valvular annulus, or aortic root dilatation (64). In a contemporary study of predominantly classic-phenotype AFD (80%), the prevalence of moderate to severe valvular disease was 10%, with mitral and tricuspid regurgitation being the most common (64). Thickening of valve leaflets and the sub-valvular apparatus are more common than severe valvular disease, affecting the mitral and aortic valve in 57% and 47% of patients, respectively (65, 66). Valvular disease is more commonly regurgitant than stenotic; in one cohort, mitral, and tricuspid regurgitation were common (15.3%, 51.4%, and 34.2% respectively), but no patients had moderate or greater aortic stenosis (65). Owing to the relative rarity of severe valvular lesions, there are few case reports of transcatheter or surgical management of severe aortic stenosis (67, 68) in AFD patients.

Aortic root dilatation, where present, is typically at the level of the sinus of Valsalva or ascending aorta. There are no contemporary estimates of the prevalence of aortic involvement; in an older cohort study of 106 AFD patients, male patients more commonly had dilatation at the level of the sinus of Valsalva (32.7% vs. 5.6%) than female patients, with a similar proportion at the ascending aorta (29.6% vs. 21.1%) (69). The prevalence of aortic dilatation increased with age, and developed at a younger age in male patients, similar to other manifestations of AFD (69). Aortic dilatation is progressive in AFD; in one study of patients followed by CMR, the mean [SD] annual expansion rate at the sinotubular junction [0.41 (0.16)mm/year] and proximal ascending aorta [0.41 (0.26) mm/year] was significantly higher than the expected progression in normal patients [0.14 (0.11)mm/year], whereas the annual growth in female patients was no greater than expected for normal patients (70). The optimal size threshold to intervene on aortic aneurysms has not been determined in AFD; acute aortic syndromes in AFD are limited to case reports (71).

## Diagnosis

Confirmatory diagnosis of AFD is dependent on α-galactosidase activity and GLA mutation analysis. In male patients, the first step is to measure α-galactosidase activity, which can be diagnostic and can be measured in leukocytes, plasma, or dried blood spot (72). Characteristically, in male patients with classic AFD, α-galactosidase activity levels are very low to undetectable (73, 74), and usually less than 3% of normal (3). By contrast, α-galactosidase activity levels are usually higher in late-onset variants of AFD, but typically less than 30% of normal (3). In one series of patients, male patients with late-onset variants of AFD had a mean α-galactosidase activity of 20% of normal, compared to 3% for male patients with the classic phenotype (5). Among female patients, due to selective X-inactivation, α-galactosidase activity has a more limited role in the diagnosis of AFD. In one study of female AFD patients using traditional reference values for α-galactosidase activity, the positive predictive value for low plasma α-galactosidase activity was 94%, with a negative predictive value of 74% (73).

GLA gene analysis is essential for both male and female patients; however, in male patients it is confirmatory after detecting low measure α-galactosidase activity, whereas in female patients, GLA analysis is typically the first step in establishing the diagnosis of AFD (73). For patients with genetic variants of unknown significance (GVUS), a detailed clinical and biochemical review for disease manifestations, a review of family history, and multidisciplinary input, particularly with a geneticist, is essential (74). Following GLA analysis in female patients, if the mutation identified does not have established pathogenicity, then measurement of lyso-Gb3 can demonstrate the clinical significance of the genetic variant of unknown significance (75–77). In one study, elevated lyso-Gb3 offered a sensitivity and specificity of 98% and 100%, respectively, for male patients, and 97% and 100% for female patients (73). By comparison; the sensitivity and specificity of α-galactosidase activity in female patients was 49% and 91%, respectively (73). More recently, the ratio between α-galactosidase activity and lyso-Gb3 has shown promise in differentiating between AFD and controls among female patients, with 100% sensitivity for differentiating AFD, compared to a sensitivity of 8.6% and 74.4% for α-galactosidase activity and lyso-Gb3 alone (78). GLA gene analysis offers disease confirmation, can be prognostic with respect to the disease manifestations associated with the specific mutation, and is important in pedigree analysis. It is also worth noting that proteinuria is an important renal manifestation of AFD, although relatively nonspecific. Detection of proteinuria can increase suspicion for AFD in the correct clinical context and is also used in follow-up to assess disease therapy.

Endomyocardial biopsy is an important modality in the diagnosis of AFD, albeit with a limited role. Endomyocardial biopsy in AFD demonstrates myocytes containing fine-granulated vacuoles positive at Sudan-black staining, indicative of lipid accumulation (116). Electron microscopy demonstrates the characteristic finding of concentric lamellar bodies composed of Gb3. Consensus guidelines on when to perform endomyocardial biopsy, or biopsy of alternative sites specifically in the context of AFD are lacking. Per the 2007 European Society of Cardiology guidelines on endomyocardial biopsy (EMB), EMB can be considered in heart failure with unexplained HCM (Class IIb) (114). Similarly, the 2021 ESC HF guidelines suggest EMB can be considered in the diagnosis of forms of cardiomyopathy where a specific treatment exists (i.e., storage diseases), where the diagnosis cannot be reached by non-invasive means (115). In light of the advances in biochemical and genetic diagnosis of AFD, EMB is usually not necessary but can be performed in cases of diagnostic uncertainty, as in the case of female patients with a late-onset phenotype with a GLA VUS.

## Investigations and imaging for cardiac involvement

### Electrocardiography

One of the earliest electrocardiographic (ECG) manifestations of AFD is shortening of the PQ interval, due to shortening of the duration of the P wave (79). Although PQ shortening is well described in AFD, it is uncommon; two separate cohort studies found a prevalence of 7.5% and 14% (80, 81). By contrast, shortening of the P_end_Q (PQ—duration of *P* wave) is more common in AFD, with one study finding a prevalence of 47.5% (80). Both QT and QRS prolongation have been described in AFD, with one study finding that QT prolongation was limited to male patients (80). The ECG findings correlate with the extent of cardiac involvement. In one study, patients with AFD but without LVH underwent CMR, and those with a low T1 signal (suggestive of glycosphingolipid deposition) had a greater maximum Q-wave amplitude, Sokolow-Lyon index, and Cornell index than AFD patients with a normal T1 signal. In another study, an ECG-based nomogram was developed to predict cardiac involvement, defined as a low T1 on CMR, using four variables: Sokolow-Lyon Index, ratio between the *P* wave and PR segment duration, QRS duration, and QT (82). Over time, patients develop ECG features of LVH, which is present in 50.5% of patients in one study (58). The ECG is valuable in the diagnosis of AFD and differentiation from mimics (Table 2): in one study, an algorithm was developed using ECG features to differentiate between AFD, hypertensive heart disease, hypertrophic cardiomyopathy, aortic stenosis, and amyloidosis (83). The combination of QTc < 440 ms with a P_end_Q < 40 ms was highly sensitive and specific for AFD (83). Additionally, an index comprised of P_end_Q, QTc, and the Sokolow-Lyon Index was able to differentiate between AFD and amyloidosis (83).

**Table 2:** Characteristic features and differentiation between causes of left ventricular hypertrophy.

Cause of LVH	ECG	Echocardiography	Cardiac MRI
Fabry disease	• Short PR (79) • Short P_end_Q (<40 ms) (80) • Greater Sokolow-Lyon index than amyloidosis (83) • QTc < 440 + P_end_Q < 40 ms sensitive and specific (83) • Inferior ST-depression more common than in sarcomeric HCM (193)	• Lower maximal wall thickness than HCM (90) • Lower ratio of septal to posterior wall thickness than HCM (84) • Low global longitudinal strain, particularly in the inferolateral wall and right ventricle relative to HCM (84) • Right ventricular systolic function less impaired than in amyloidosis for the same degree of right ventricular hypertrophy (93)	• Predominantly concentric LVH (194) • Late gadolinium enhancement, particularly of the inferolateral wall (194) • Low T1 due to glycosphingolipid deposition (195, 196), not seen in other causes of HCM (197) • Pseudonormalization of T1 in regions of LGE (195, 196) • Reduced perfusion, particularly in areas with LVH, low T1, or LGE (95) • ECV generally unchanged (198)
Cardiac amyloidosis	• Low voltage in limb leads (<0.5 mV) (199) • Pseudo-infarct pattern (Q waves on 2 contiguous leads without coronary artery disease) (199)	• More severe diastolic dysfunction (E/e′) relative to AFD (200) and sarcomeric HCM (201) • Apical sparing left ventricular strain pattern (202) • Lower global longitudinal, circumferential, and radial strain than sarcomeric HCM and hypertensive HCM (201) • EFSR index highly sensitive (89.7%) and specific (91.7%) (201) • More likely to have a pericardial effusion than other causes of HCM (203) • More likely to have a granular sparkling myocardial appearance (201, 204)	• T1 is significantly higher than in sarcomeric HCM (205), hypertensive HCM (206), and AFD (197) • LGE is characteristically global subendocardial (206) • ECV is significantly increased, greater than in AFD or sarcomeric HCM (198)
Sarcomeric hypertrophic cardiomyopathy	• LVH by Sokolow-Lyon criteria, deep T-wave inversion in inferior and lateral leads, pathologic Q waves commonly described (207) • QRS less prolonged than AFD, and less likely to have right bundle branch block or non-specific intraventricular conduction delay than AFD (193)	• Hypertrophy may be septal-predominant, apical-predominant, mid-ventricular, or symmetric (208) • Mid-cavitary or outflow tract obstruction may be seen, and are more common than in hypertensive HCM (209) • Diastolic dysfunction, left atrial enlargement are more severe with greater LV mass than in hypertensive HCM (210) • Apical-basal strain gradient preserved relative to amyloidosis (202) • LVH asymmetric relative to hypertensive HCM (211) and AFD (84)	• LGE is patchy, multifocal, and commonly seen at the RV insertion point (212) and the mid-wall of hypertrophied segments (213) • ECV is higher than in hypertensive HCM (214) and AFD, but lower than amyloidosis (198)
Hypertensive heart disease	• Multiple criteria described for LVH, commonly Sokolow-Lyon (215)	• More likely to have concentric pattern of LVH, but asymmetric septal hypertrophy common (210)	• Left ventricular end-diastolic wall thickness generally <15 mm (216) • LGE if present, is in non-specific pattern (217) • T1 and ECV may be normal; if increased less than other causes of HCM (216, 217)

AFD, Anderson-Fabry disease; ECV, Extracellular volume; EFSR, Ejection fraction to global longitudinal strain ratio; HCM, hypertrophic cardiomyopathy; LGE, late gadolinium enhancement; LVH, left ventricular hypertrophy; P_end_Q, PQ interval—duration of *P* wave; QTc, Corrected QT interval.

### Echocardiography

**Figure 2:**
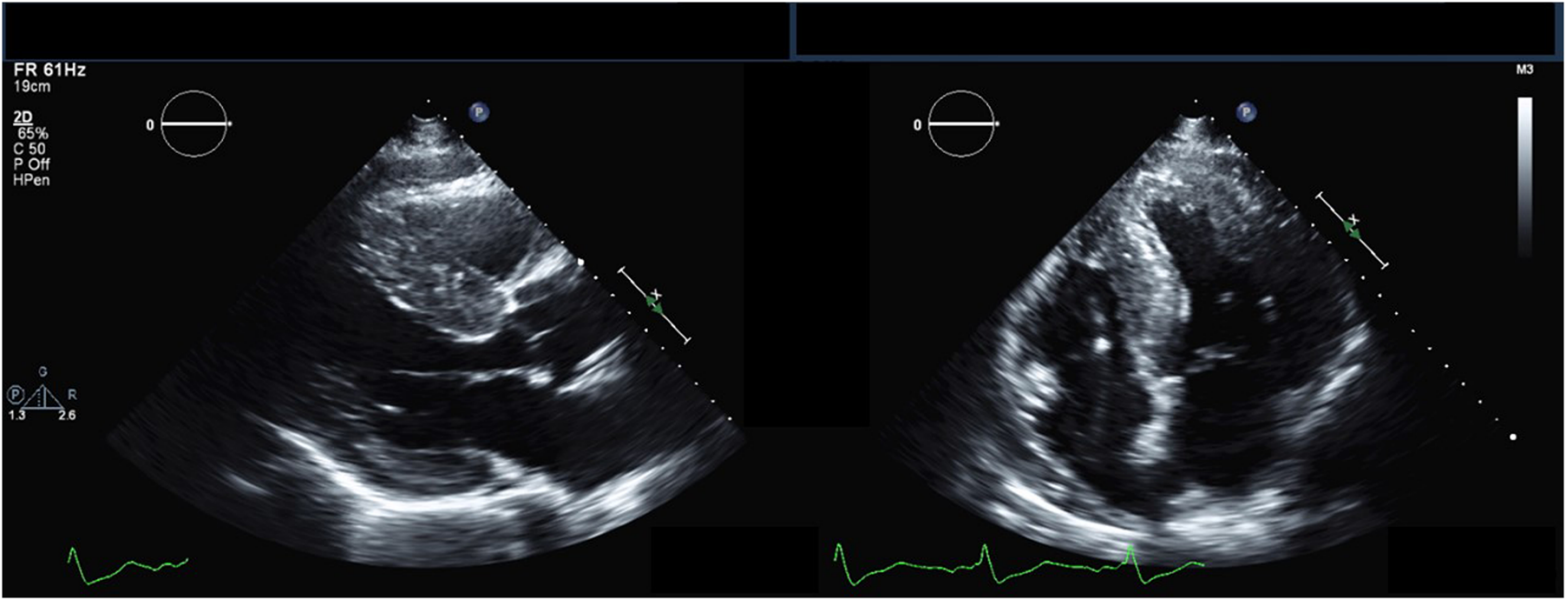
Echocardiography findings in Anderson-Fabry disease. Parasternal long-axis (left) and apical 4-chamber (right) transthoracic echocardiography views demonstrating increased left ventricular wall thickness.

The hallmark echocardiographic abnormality of AFD is LVH, with or without diastolic dysfunction (Figure 2) (84). The binary sign, previously thought to be specific to AFD, is the finding of a hyperechogenic endocardial layer adjacent to a hypoechogenic subendocardial layer, due to relative sparing of the outer myocardium from glycosphingolipid deposition (85). Subsequent analyses have found this to be insensitive and non-specific for AFD (86, 87).

In an effort to identify AFD prior to the develop of overt LVH, a variety of techniques have been utilized to identify early myocardial involvement on echocardiography. Tissue doppler imaging allows for characterization of diastolic function and has been found to be abnormal prior to the development of LVH. In one study, patients with AFD but without LVH had a lower early diastolic mitral valve inflow (Ea) and a shorter isovolumic relaxation time than controls (88). Patients with LVH had a significantly longer isovolumic relaxation time, with a reduced systolic myocardial velocity, which was negatively correlated with septal and posterior wall thickness (88). Another older study had shown abnormalities in mitral annulus e′ even in the absence of LVH (89); this was not found in a larger, contemporary study (58). Strain imaging using speckle-tracking echocardiography, a newer technique, can allow for earlier identification of abnormalities in systolic and diastolic function in both ventricular and atrial function. In the same study of 160 patients with AFD with or without LVH, GLS was abnormal in both LVH+ and LVH- patients, with regional differences noted, and a correlation between GLS and LV mass index (58). LVH+ had the lowest longitudinal strain among all segments; however, compared to controls, LVH- patients had lower peak longitudinal strain in the apical, mid, anterior, and inferolateral segments (58). Speckle-tracking echocardiography has also been used to assess atrial function in AFD; patients with AFD have a reduction in all three phasic functions of the left atrium: reservoir, conduit, and contractile function (55).

Echocardiography has a role in differentiating between AFD and other causes of LVH (Table 2). In one study comparing the echocardiograms of patients with AFD and hypertrophic cardiomyopathy (HCM), patients with AFD had a significantly lower maximal wall thickness, and greater indexed sinus of Valsalva diameter (90). In another study, compared to patients with HCM, patients with AFD had a significantly lower interventricular septum to posterior wall thickness ratio, and had worse regional longitudinal strain in the inferolateral left ventricular wall and right ventricular free wall (84). Both right ventricular free wall strain and GLS are significantly more impaired in AFD than HCM (91). By contrast, a study of left atrial function found no differences in left atrial reservoir, conduit, and contractile function between AFD and HCM patients, although HCM patients had significantly larger left atria (92). Echocardiography has also been used to compare AFD and cardiac amyloidosis; compared to patients with cardiac amyloidosis, patients with AFD have relatively preserved right ventricular systolic function at the same degree of right ventricular hypertrophy, with higher TAPSE, right ventricular S′, and right ventricular fractional shortening (93).

### Cardiac magnetic resonance imaging

**Figure 3:**
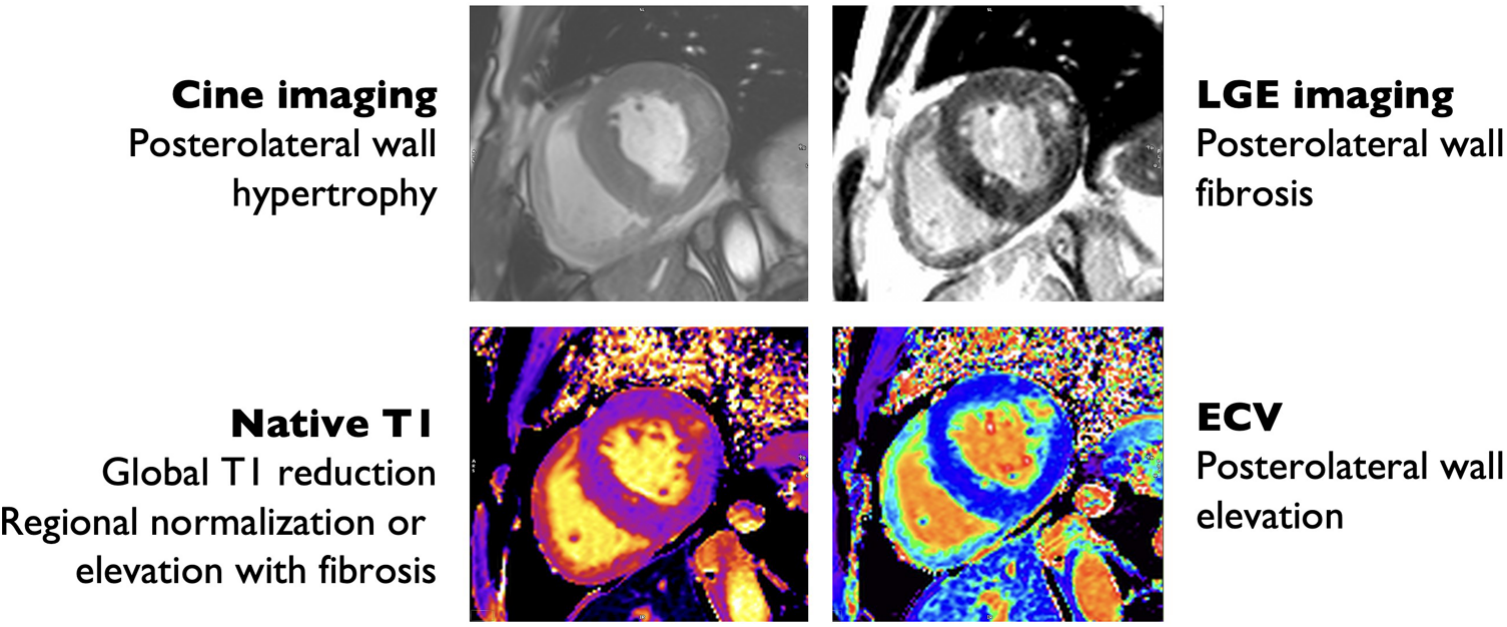
Cardiac magnetic resonance imaging findings in Anderson-Fabry disease. Hypertrophic with prominent hypertrophy of the posterolateral and lateral wall (top left) with posterolateral wall replacement fibrosis by late gadolinium enhancement (LGE) imaging (top right), also evident by increased extra-cellular volume (ECV) quantification (bottom right). There is reduced interventricular septum native T1 time consistent with glycosphingolipid deposition (bottom left).

Cardiac MRI is a comprehensive diagnostic modality that allows for the assessment of a variety of cardiac sequelae of AFD, including LVH, particularly with papillary muscle involvement; subtle abnormalities in systolic function, sphingolipid deposition, and fibrosis. Patients with AFD commonly have enlarged atria and reduced left atrial strain prior to the onset of LVH, representing an atrial myopathy that correlates with disease severity (94). Perfusion is often abnormal, particularly in areas with LVH, low T1, or late gadolinium enhancement (LGE) (95). The pattern of LGE in AFD is often non-specific, but most commonly involves the basal posterior wall and corresponds with areas of collagen deposition and fibrosis (Figure 3) (96). The presence of LGE is associated with increased risk of cardiac events, with the highest risk among patients with LGE > 15% of left ventricular mass (97). LGE is correlated with elevated troponin in AFD, which is persistently elevated in up to 21% of patients (111, 112).

Cardiac MRI is the gold-standard for the assessment of left ventricular mass index (LVMI) and maximal wall thickness (MWT), and offers greater inter-rater reliability than echocardiography (98). In one study comparing CMR and echocardiography in patients with AFD, LVMI and MWT were higher on echocardiography, with discrepant measurements between echocardiography and cardiac MRI in 26% (98). As a result, 26% of patients had conflicting recommendations regarding whether to start ERT (98). In addition to providing comprehensive assessment of cardiac involvement in AFD, CMR can also be used to differentiate between causes of cardiomyopathy, and assess the clinical stage of AFD.

T1 is particularly useful for differentiating between AFD and other causes of cardiomyopathy (Figure 3). T1 is low in the setting of fat infiltration (arrhythmogenic RV dysplasia), glycosphingolipid infiltration (AFD), and iron deposition (hemochromatosis), and high in the setting of fibrosis or amyloid protein deposition (99). T1-mapping has previously been used to differentiate between AFD and cardiac amyloidosis and HCM (100, 101). AFD can be differentiated from iron deposition using T2*-weighted imaging (99). An important limitation to the use of T1-mapping is pseudo-normalization of T1 due to the combination of glycosphingolipid deposition and fibrosis (102). Pseudo-normalization can be suspected when T1 values are normal in areas with late gadolinium enhancement (LGE) (102).

A novel application of cardiac MRI is in disease staging. Cardiac MRI has identified four phenotypes of cardiac involvement in AFD: a pre-deposition phase, where sphingolipid deposition is undetectable; a detectable accumulation phase; a hypertrophic phase; and a fibrosis phase (103). On cardiac MRI, the detectable deposition phase can be identified by a low T1 value. However, patients with AFD in the pre-detectable deposition phase have observable abnormalities on cardiac MRI, including reduced GLS and myocardial blood flow (103). In one cohort study of pre-hypertrophic AFD, 59% of patients with AFD but without LVH had a low T1 value compared to 0% of healthy controls, which was associated with the presence of LGE and ECG abnormalities (104). Although MWT and LVMI were normal in AFD patients, subtle abnormalities were present, including a greater papillary muscle mass and longer anterior mitral valve leaflet (104). Over the natural history of AFD, T1 decreases due to glycosphingolipid deposition, and then begins to stabilize in female and increase in male patients with LVH (105). The increase in T1 later in the course of illness can be explained in part by fibrosis, as well as dilution due to the normal T1 of hypertrophied myocardium, as only a minority of LVH in AFD is caused by glycosphingolipid deposition (16, 111). Cardiac MRI offers promise in identifying patients with very early cardiac involvement, who may derive the greatest benefit from early pharmacologic therapy.

### Other cardiac imaging modalities

A variety of modalities have been used to assess myocardial perfusion in AFD, including CT perfusion (80), SPECT (106), and CMR (95). Perfusion abnormalities are generally attributed to LVH and small vessel ischemia (20, 113). On perfusion CMR, myocardial blood flow is correlated with wall thickness, T2, LGE, and extracellular volume (95). The use of positron-emission tomography (PET) in AFD is increasingly being described (107, 108). Increased uptake in the basal inferolateral wall in AFD on PET has been well-documented, similar to the distribution of LGE on CMR (107, 109). In one study of hybrid PET-CMR, patients with focal 18F-FDG uptake had higher T1 values, suggestive of pseudo-normalization (108). PET has also been used to evaluate other causes of cardiomyopathy such as amyloidosis and sarcoidosis (110); however, features on PET al.lowing for the differentiation of cardiomyopathies have yet to be identified. PET has also been used to characterize the perfusion abnormalities in AFD; in one cohort, coronary microvascular dysfunction was evident in all AFD patients, with the greatest reduction in myocardial blood flow in patients with LVH (14). Perfusion abnormalities were global, with a marked reduction in myocardial blood flow at the apical segments (14). PET, and PET-CMR may allow for earlier recognition of cardiac involvement in AFD as evidenced by reduced perfusion prior to the development of overt LVH, and identify early fibrosis (108).

## Management

### Enzyme replacement therapy

The mainstay of therapy for AFD is enzyme replacement therapy. There are two formulations available: agalsidase-alfa, derived from human fibroblasts, and agalsidase-beta, derived from hamster ovarian cells. In the United States, only agalsidase-beta is licensed, whereas both formulations have been licensed for use in Canada and throughout Europe (117). Both formulations are structurally similar and thought to have a similar efficacy. Although a systematic review of observational cohort studies suggested a significantly lower incidence of cerebrovascular events in patients treated with agalsidase-alfa than those treated with agalsidase-beta (118), a Cochrane review of randomized trial data found no difference in renal deterioration, cardiac events, death, acroparesthesia, or reduction in Gb3 levels in patients treated with agalsidase-alfa compared to agalsidase-beta (119). Administration of ERT is generally well tolerated; pertinent adverse effects include infusion reactions and development of anti-drug antibodies (ADAs).

Given that ERT is comprised of a foreign recombinant protein, a humoral response leading to the development of ADAs is common, generally within 3–6 months of treatment initiation (120, 121). Once ADAs develop, they are generally thought to be permanent (122, 123). Up to 40% of male patients treated with ERT develop ADAs; patients with minimal or absent production of α-galactosidase are at particularly high risk as there is no endogenous α-galactosidase for the immune system to develop tolerance to (120). In one observational study, patients treated with agalsidase-beta had a greater risk of developing ADAs than those treated with agalsidase-alfa (52% vs. 28% of male patients), which may relate to the greater dose of agalsidase-beta administered (1 mg/kg every 2 weeks for agalsidase beta compared to 0.2 mg/kg every 2 weeks for agalsidase alfa) (124). The risk of antibody development also appears to be higher in those with a more severe, classic disease phenotype (125), and patients with a higher Gb3 level at treatment initiation (126). Antibodies to either formulation are thought to be cross-reactive, such that switching when antibodies develop may be of little benefit (121). However, the clinical significance of the development of ADAs is unclear. In one study, patients with ADAs had higher Gb3 levels than those treated with ERT but without antibodies, however there was no difference in the progression of renal impairment (125). In a separate retrospective study comparing patients with or without ADAs found that antibody-positive patients had a deterioration in renal function, increased MSSI and disease severity index, whereas disease activity remained stable in the antibody-negative group (123). The optimal strategy for monitoring for the development of ADAs is unclear, as well as how to respond to the development of ADAs. ERT can be dosed to achieve saturation of ADAs; in one observational study, compared to patients who achieved saturation of ADAs, those that did not had a greater decline in eGFR, an increase in interventricular septal thickness, and a lesser reduction in lyso-Gb3 (127).

Existing guidelines emphasize the early introduction of ERT, prior to the development of irreversible end-stage organ damage. In both the Canadian guidelines and European Fabry Working Group, patients with renal (elevated creatinine, proteinuria), cardiac (LV mass, septal thickness, arrhythmia), neurologic (stroke, hearing loss, pain), or gastrointestinal involvement qualify for initiation of ERT (128, 129). The European guidelines are less conservative, also advising initiating ERT in all male patients with classical AFD older than 16 years prior to the development of symptoms or organ involvement (129). Both guidelines also address which patients should not initiate ERT, and in whom therapy can be discontinued. Patients with end-stage renal disease who are not transplant candidates, patients with advanced HF, patients with significant myocardial fibrosis, and those with comorbid conditions with a life-expectancy less than 1 year who are not expected to derive a clinical or quality of life benefit from treatment (128, 129).

Enzyme replacement therapy with agalsidase-alfa or -beta has demonstrated efficacy in the reduction of Gb3 and improvement in cardiac, renal, and neurologic outcomes. Treatment with ERT has been shown to reduce plasma and urinary Gb3 and lyso-Gb3, a deacetylated form of Gb3 (7, 129–132). Treatment with ERT offers benefit for both male and female patients, as well as those with classical and late-onset AFD phenotypes. In a meta-analysis of both randomized and observational trials that separated results by sex, ERT led to stabilization in LVMI in male patients with AFD and LVH at baseline; among male patients without LVH at baseline, the increase in LVMI over time was lower ERT-treated patients (133). Among female patients, ERT led to a reduction in LVMI among patients with LVH at baseline, and stabilization of the LVMI among female patients without LVH at baseline (133). A more recent systematic review and pooled analysis that included a balance of male and female patients found a reduction in LVMI with ERT (134). Early initiation of treatment is paramount; patients with existing LGE on CMR are unlikely to experience regression of LVH with ERT (135, 136). In a study comparing patients with AFD who started ERT within 24 months of diagnosis and those who started after 24 months, earlier initiation of ERT was associated with reduced risk of cardiovascular events, defined as HF, arrhythmia, cardiac surgery, LVH, conduction abnormality, and myocardial infarction (137).

### Advancements in enzyme-replacement therapy

Treatment with ERT is limited by its short half-life, immunogenicity, and limited tissue penetration. Novel therapies for AFD aim to address these shortcomings. Pegunigalsidase-alfa is a pegylated, covalently cross-linked form of agalsidase-alfa whose role in the treatment of AFD is being evaluated in ongoing trials (NCT03180840, NCT04552691). The pegylated, cross-linked structure is thought to increase plasma-half life, and reduce immunogenicity (138). In one study, pre-existing ADAs to agalsidase-alfa or -beta demonstrated reduced affinity for pegunigalsidase-alfa, with a 30% reduction in inhibitory capacity (139). In a small study of 18 patients, patients treated with pegunigalsidase-alfa exhibited stability of renal function over the year of treatment (140). The effect of pegunigalsidase-alfa on cardiac outcomes has yet to be determined. Another novel agent is moss-derived agalsidase-alfa (Moss aGal). Moss-derived agalsidase-alfa differs from traditional ERT in its glycosylation profile and cellular uptake, relying on mannose receptors rather than mannose-6-phosphate receptor-mediated endocytosis (141). Mannose receptors are expressed on vascular endothelial, smooth muscle, dendritic and renal mesangial cells, making Moss-aGal attractive for treatment of AFD, and may help overcome limitations in the tissue distribution of ERT (141). Treatment with Moss-aGal has been shown to reduce urinary Gb3 and is well-tolerated, however the effect on hard clinical outcomes has not been established (141). In a mouse model of AFD, treatment with Moss-aGal led to similar distribution in the heart and spleen compared to agalsidase-alfa treated mice, with better distribution in the kidney, and similar efficacy for reduction in Gb3 (142). The risk of ADA development with Moss-aGal has not previously been explored owing to the preclinical nature of Moss-aGal trials, however, ADAs to agalsidase-alfa or -beta have similar affinity to Moss-aGal (143).

### Chaperone therapy

Chaperone therapy is alternative to ERT for the treatment of AFD that aims to correct misfolded α-galactosidase and improve its stability, allowing it to be delivered to the lysosomes, where it can degrade Gb3 (144). Chaperone therapy is only effective in amenable mutations where unstable but functional α-galactosidase is produced; mutations resulting in the absence of production of α-galactosidase would not be amenable to chaperone therapy. Missense mutations in particular may respond to treatment with chaperone therapy, whereas large deletions, frameshift mutations, splicing mutations, and insertions are not responsive (144). In-vitro assays of responsiveness should be performed prior to initiation of treatment, defined as an increase in α-galactosidase activity ≥1.2-fold, or an absolute α-galactosidase activity of ≥3% of normal (145).

Migalastat is the only chaperone agent that is currently licensed for the treatment of AFD. The ATTRACT trial, which randomized patients with AFD to continue ERT or switch to migalastat found that migalastat, but not ERT led to a reduction in LVMI; among patients with existing LVH, the effect size was greatest (146). The composite of renal (worsening of eGFR, proteinuria), cardiac (myocardial infarction, arrhythmia, HF, unstable angina) and cerebrovascular events (stroke, transient ischemic attack) was numerically but not significantly lower among patients treated with migalastat (29% vs. 44%, *p* = 0.36) (146). Similarly, the FACETS trial found that patients treated with migalastat experienced a significant reduction in LVMI at 24 months, particularly among patients with LVH at baseline (147). In the more recent MAIORA study, an uncontrolled before-after study of treatment-naïve AFD patients, migalastat was associated with stabilization of LVMI at 18 months, a trend towards increased septal T1 on cardiac MRI, and improved exercise tolerance (148). As is often seen with ERT, patients with the earliest stage of disease derived the greatest benefit from migalastat, emphasizing the role of early treatment for AFD (148). Chaperone therapy has the advantages of oral administration and less immunogenicity than ERT but is only effective in a subset of patients. Observational studies have also demonstrated a reduction in LVMI with migalastat, including in patients switched from ERT to migalastat (149–151).

### Novel therapies

Novel agents have been developed and are now available for the treatment of AFD that do not rely on enzyme replacement. Substrate reduction therapy (SRT) is one such method that involves reducing the production of Gb3 through the inhibition of glucosylceramide synthase, an enzyme involved in the production of glycosphingolipids. SRT has established efficacy in Gaucher disease, another glycogen storage disease (152). Three agents have been studied in AFD: eliglustat, venglustat and lucerastat. In a mouse model of AFD, treatment with eliglustat reduced levels of Gb3, and was more effective than ERT in reducing levels of Gb3 in the kidney, but less effective at reducing Gb3 in the heart or liver (153). The combination of SRT and ERT was most effective at reducing Gb3 in all tissues (153). Similarly, venglustat has been shown to reduce levels of Gb3 and lyso-Gb3 in a mouse model of AFD, and unlike ERT, lowered Gb3 and lyso-Gb3 in the brain (154). In a small study of 10 patients receiving ERT with lucerastat and 4 patients receiving ERT alone, the combination led to a greater decrease in plasma Gb3 and urinary Gb3, but no change in LVEF or LVMI at 12 weeks (155). While larger studies with longer follow-up are needed, SRT is promising as an addition to ERT given its tolerability, oral route of administration, lack of anti-drug antibodies, and ability to cross the blood brain barrier (153). Trials are underway to determine the efficacy of SRT monotherapy in AFD (NCT03425539, NCT05280548).

Another advance in the treatment of AFD is mRNA therapy encoding recombinant α-galactosidase. In a mouse model of AFD, mRNA therapy led to a significant reduction in Gb3 in cardiac and renal tissue, with similar efficacy to ERT over two months of treatment (156). Administration of mRNA to non-human primates has been shown to increase the activity of α-galactosidase in the heart, liver, and spleen, but not kidney (157). The effect of mRNA therapy in AFD patients has yet to be established. Another investigational treatment for AFD is gene therapy which can take several forms: direct administration of adenovirus/adeno-associated virus containing GLA DNA to the target organ; infusion and engraftment of autologous hematopoietic stem/progenitor cells with lentivirus-mediated transduction of GLA DNA into host DNA; and gene editing with CRISPR/Cas systems (158). Studies involving the administration of lentivirus-transduced hematopoietic stem/progenitor cells to a limited number of patients with AFD have demonstrated sustained increases in α-galactosidase activity; large-scale studies with hard clinical endpoints are needed (159, 160).

### Management of cardiovascular complications

**Figure 4:**
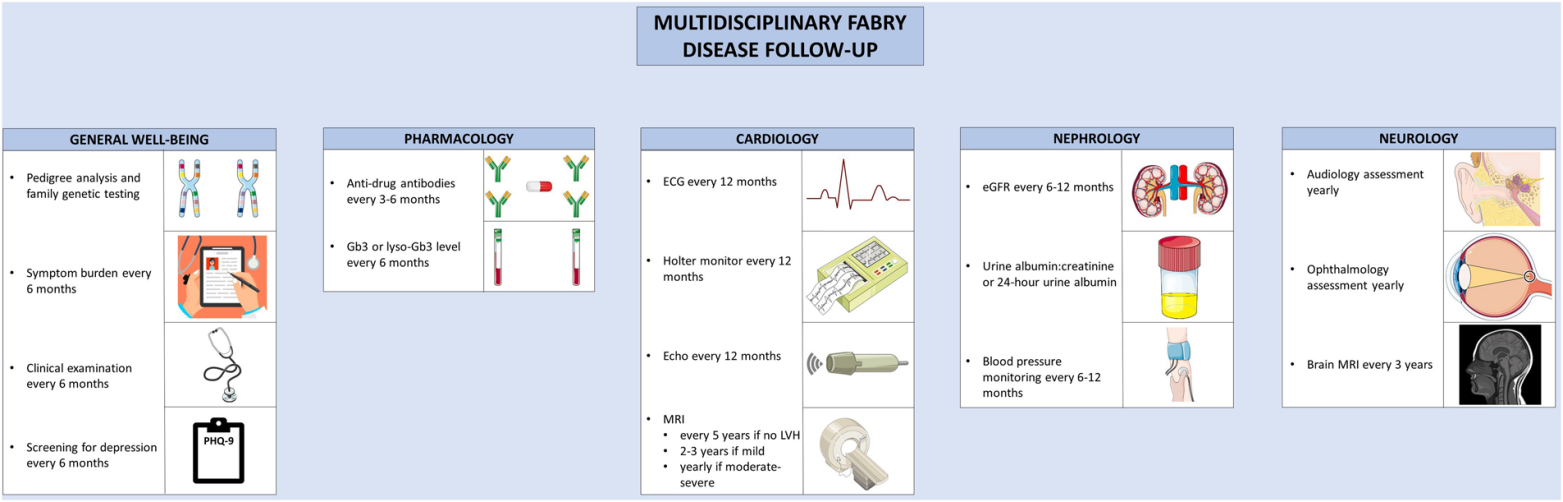
Multidisciplinary follow-up for Anderson-Fabry disease. Recommended follow-up for Anderson-Fabry disease involves co-ordinated care between disciplines. A variety of clinical, biochemical, and imaging-based techniques are used to screen for complications. The figure was partly generated using Servier Medical Art, provided by Servier, licensed under a Creative Commons Attribution 3.0 unported license, as well as from freepik.com (https://www.freepik.com/free-vector/doctor-writing-clipboard_2095625.htm.

Owing to the myriad of organ systems involved in AFD, multidisciplinary care is essential to manage the complications (Figure 4). Multidisciplinary care models have been developed for the care of AFD patients, typically involving a geneticist, nephrologist, cardiologist, neurologist, nurse specialists, and psychologists (161, 162). Hypertension is common in AFD; and in one cohort, was uncontrolled in 57% of male and 47% of female patients (163). Given the prevalence of proteinuria and renal dysfunction in AFD, ACE-inhibitors or angiotensin receptor blockers should be considered for the treatment of hypertension (164, 165). In one study, proteinuria was present in 91% of male and 72% of female patients with AFD and eGFR < 60 ml/min/1.73 m^2^ (166). Beta-blockers or non-dihydropyridine calcium channel blockers should be used with caution in the presence of conduction system disease (165). HF in AFD should be managed according to standard guidelines; beta-blockers and ivabradine should be used with caution given the prevalence of conduction disease in AFD (167). Similarly, the management of atrial fibrillation or flutter are similar in AFD and the general population, with the caveat that amiodarone should generally be avoided given its ability to alter lysosomal pH, thereby reducing the activity of endogenous and exogenous α-galactosidase (168). Additionally, anticoagulation is recommended in all patients with atrial fibrillation or flutter where the bleeding risk is not prohibitive (167). Furthermore, maintenance of sinus rhythm over a rate control strategy is recommended (169). Bradyarrhythmias requiring anti-bradycardia pacing are common; patients with prolonged PR or QRS should be monitored closely (170). Proposed indications for the insertion of an implantable cardioverter-defibrillator mirror that of hypertrophic cardiomyopathy; for patients with unexplained syncope, sustained ventricular fibrillatory, cardiac arrest, non-sustained ventricular tachycardia, and extensive cardiac fibrosis (169, 171). Where possible, patients with AFD should be referred to centers of excellence that allow for comprehensive care in multidisciplinary clinics.

## Follow-up monitoring and surveillance

Guidelines have been developed for the routine clinical follow-up of patients with AFD (169). Routine follow-up for patients with AFD involves monitoring for treatment efficacy and disease progression. Patients should be monitored for symptoms of AFD, ideally using a validated assessment tool; the Brief Pain Inventory, EQ-5D, or MSSI can be used to assess pain, quality of life, and overall disease activity, respectively (172). Clinical examination and assessment of symptom burden should be performed every 6 months (172). To assess treatment efficacy, glycolipid burden should be monitored with lyso-Gb3 or Gb3 levels, performed at baseline, every 6 months, and after treatment switches (169). ADAs should be tested for at baseline, every 3–6 months during the first 18 months of therapy, and then every 6 months thereafter (132, 169). Echocardiography should be performed at diagnosis, at the time of development of new or worsening symptoms, and annually thereafter. In the presence of exertional intolerance, exercise echocardiography can be performed to evaluate for provoked left ventricular outflow tract obstruction or mitral regurgitation, and cardiopulmonary exercise testing or treadmill ECG can be performed to assess for chronotropic incompetence (169). Where possible, CMR should be performed in all adult patients at the time of the initial evaluation (169). If the initial CMR is normal, it can be repeated every 2–5 years (169, 172). In the presence of LVH, the CMR should be repeated every 2–3 years if mild, or annually in the presence of moderate to severe LVH (169, 172). Yearly ECG and 24 h Holter monitoring should be performed to assess the burden of arrhythmia (171). For patients with unexplained palpitations or left atrial enlargement, more frequent 48-hour monitoring should be considered. In the case of unexplained syncope, longer-term monitoring with an implantable loop recorder should be considered (169). The follow-up and surveillance of AFD and its diverse manifestations can be better coordinated in specialized disease-management clinic, where available.

## Conclusions

AFD is an X-linked, heritable disease caused by mutation of GLA, leading to reduced production of α-galactosidase, and the accumulation of glycosphingolipids. There is no one characteristic mutation causing AFD; a variety have been described with varying phenotypes. Patients can present with classic disease, characterized by early onset of peripheral neuropathy, LVH, and proteinuric chronic kidney disease; or may present with a late-onset, cardiac-predominant phenotype. Patients with AFD face a significant burden of cardiovascular involvement, including HF, microvascular ischemia, brady- and tachyarrhythmias, valvular dysfunction, and aortic dilatation. The diagnosis of AFD relies on multidisciplinary input, and involves assessment of α-galactosidase activity, measurement of glycosphingolipids, and GLA mutation testing. Non-invasive modalities such as echocardiography and CMR can be used to assess the burden of disease and monitor response to therapy. The backbone of contemporary therapy is ERT; chaperone therapy represents an alternative for patients with amenable mutations. Novel forms of enzyme replacement therapy have been developed to address concerns of immunogenicity, tissue penetration, and duration; however, larger clinical trials are needed. A variety of experimental therapies have been developed for AFD, including substrate reduction therapy, mRNA therapy, and gene therapy, with ongoing randomized clinical trials to establish their role in the care of AFD patients. Long-term, multi-disciplinary care is needed to address the multi-organ complications of AFD and monitor disease activity.
